# A method for dynamic subtraction MR imaging of the liver

**DOI:** 10.1186/1471-2342-6-5

**Published:** 2006-06-07

**Authors:** Luca T Mainardi, Katia M Passera, Agnese Lucesoli, Paolo Potepan, Ernesto Setti, Renato Musumeci

**Affiliations:** 1 Dipartimento di Ingegneria Biomedica, Politecnico di Milano, Italy; 2 Dipartimento di Elettromagnetismo e Bioingegneria, Università Politecnica delle Marche, Italy; 3 Dipartimento di Diagnostica per Immagini e Radioterapia, Istituto Nazionale per la Cura e la Prevenzione dei tumori di Milano, Italy; 4 Laboratorio di Analisi Radiologica Avanzata (LARA), Milano, Italy

## Abstract

**Background:**

Subtraction of Dynamic Contrast-Enhanced 3D Magnetic Resonance (DCE-MR) volumes can result in images that depict and accurately characterize a variety of liver lesions. However, the diagnostic utility of subtraction images depends on the extent of co-registration between non-enhanced and enhanced volumes. Movement of liver structures during acquisition must be corrected prior to subtraction. Currently available methods are computer intensive. We report a new method for the dynamic subtraction of MR liver images that does not require excessive computer time.

**Methods:**

Nineteen consecutive patients (median age 45 years; range 37–67) were evaluated by VIBE T1-weighted sequences (TR 5.2 ms, TE 2.6 ms, flip angle 20°, slice thickness 1.5 mm) acquired before and 45s after contrast injection. Acquisition parameters were optimized for best portal system enhancement. Pre and post-contrast liver volumes were realigned using our 3D registration method which combines: (a) rigid 3D translation using maximization of normalized mutual information (NMI), and (b) fast 2D non-rigid registration which employs a complex discrete wavelet transform algorithm to maximize pixel phase correlation and perform multiresolution analysis. Registration performance was assessed quantitatively by NMI.

**Results:**

The new registration procedure was able to realign liver structures in all 19 patients. NMI increased by about 8% after rigid registration (native vs. rigid registration 0.073 ± 0.031 vs. 0.078 ± 0.031, n.s., paired *t*-test) and by a further 23% (0.096 ± 0.035 vs. 0.078 ± 0.031, p < 0.001, paired *t*-test) after non-rigid realignment. The overall average NMI increase was 31%.

**Conclusion:**

This new method for realigning dynamic contrast-enhanced 3D MR volumes of liver leads to subtraction images that enhance diagnostic possibilities for liver lesions.

## Background

Dynamic Contrast-Enhanced 3D Magnetic Resonance Imaging (DCE-MRI) detects and accurately characterizes many types of liver lesion [1-8]. Volumetric Interpolated Breath-hold Examination (VIBE) sequences yield images of the entire abdomen with high spatial resolution, nearly isotropic voxel size (2 mm or less) and short acquisition time (<25s) [9-11]. In DCE-MRI, the liver is imaged during breath-hold (reducing respiratory motion artifacts) before and after the injection of contrast in selected phases of contrast distribution (typically early and late hepatic arterial phases and portal venous phase). Imaging the portal phase is important for detecting and characterizing hypovascular lesions and metastatic deposits, particularly colon cancer secondary lesions [1,4,5].

The application of a technique to subtract pre- from post-contrast images [12-14] makes it easier to pick out and characterize lesions, by eliminating common background signals and emphasizing hyper (or hypo) enhanced structures. However, subtraction is not a trivial operation: liver structures may move or deform between the two acquisitions and a registration procedure is required to realign structures prior to subtraction [12]. In a study where subtraction was applied without registration, the resulting image was not diagnostic for most (73%) subcentrimetic lesions [13]. Various algorithms have been proposed for the registration of liver volumes, and applied to MRI-guided ablation of liver cancer [15], the planning of single-dose radiation therapy [16-18] and liver surgery [19]. Alignment by maximization of mutual information (MI) [23-25] is usually performed. In one study [15], MI was found to be the best of five methods for the registration of DCE-MR images of the liver.

The registration strategy must also take into account the fact that the main component of liver motion is a global cranio-caudal excursion, due to diaphragm movement during respiration [31]. Deformations may also be important since liver is a soft tissue [32,33]. Although solely rigid registration methods have been proposed [15,16], the current trend is towards registration strategies that combine rigid and non-rigid approaches [17,20]; however maximization of MI in a non-rigid framework requires long processing times that may limit clinical application.

We report a new 3D registration method for the dynamic subtraction of volumetric liver images, that combines rigid and non-rigid approaches, and yet is light on computational cost. Rigid registration was obtained by volume translation, maximizing normalized MI between two volumes, while local 2D non-rigid realignment employed a Complex Discrete Wavelet Transform (CDWT) algorithm. The CDWT algorithm we used has been applied to the registration of MR images of the breast [28] where it has been shown to be fast, robust and reliable [27]. We present and discuss the application of this method for characterizing liver lesions during portal phases.

## Methods

### Experimental protocol and image acquisition

The algorithm was tested in an experimental protocol developed at the Department of Images for Diagnosis and Therapy of the National Cancer Institute of Milan and carried out September through November 2004. We examined 19 consecutive patients of median age 45 years (range 37–67) with suspected primary or metastatic liver parenchyma lesions following US or CT examination. All patients underwent 1.5 T MR imaging (Siemens Vision, Erlangen). VIBE T1-weighted sequences (TR 5.2 ms, TE 2.6 ms, flip angle 20°, slice thickness 1.5 mm) were acquired (axial orientation) before and 45s after contrast agent injection. Acquisition parameters were optimized to best enhance the portal system; 70 to 112 slices per volume were acquired depending on liver size. Breath-holds were at exhale and lasted 21–26s. Gd-DTPA and Gd-BOPTA were used as contrast agents: 0.2 mmol/kg was injected in a single bolus at 1.5–2 ml/s followed by 20 ml physiologic saline (0.09% NaCl) at the same rate to accelerate contrast dissemination to the central venous system.

### Image preprocessing

To improve image quality before registration, including reducing image noise and liver segmentation, the following pre-processing steps were applied.

Volumetric acquisitions have lower signal-to-noise ratio than 2D acquisitions [29]. Superimposed noise may reduce the efficiency of registration [27] and image noise is amplified by volume subtraction thereby reducing the possibilities for structure detection and tissue characterization. Classical noise filtering techniques introduce blurring [30] and reduce spatial resolution and are therefore not suitable. To attenuate noise while preserving image details, an adaptive filter designed for brain MR images was used [30]. Briefly, the filter works as follows: for each pixel (*x*,*y*), a limited homogeneous region enclosing the pixel (template) was searched. The filter output *T*(*x*,*y*) was calculated using the following adaptive 2D formula:

T(x,y)=σk2(x,y)I(x,y)+σn2mjσk2(x,y)+σn2,
 MathType@MTEF@5@5@+=feaafiart1ev1aaatCvAUfKttLearuWrP9MDH5MBPbIqV92AaeXatLxBI9gBaebbnrfifHhDYfgasaacH8akY=wiFfYdH8Gipec8Eeeu0xXdbba9frFj0=OqFfea0dXdd9vqai=hGuQ8kuc9pgc9s8qqaq=dirpe0xb9q8qiLsFr0=vr0=vr0dc8meaabaqaciaacaGaaeqabaqabeGadaaakeaacqWGubavcqGGOaakcqWG4baEcqGGSaalcqWG5bqEcqGGPaqkcqGH9aqpdaWcaaqaaGGaciab=n8aZnaaDaaaleaacqWGRbWAaeaacqaIYaGmaaGccqGGOaakcqWG4baEcqGGSaalcqWG5bqEcqGGPaqkcqWGjbqscqGGOaakcqWG4baEcqGGSaalcqWG5bqEcqGGPaqkcqGHRaWkcqWFdpWCdaqhaaWcbaGaemOBa4gabaGaeGOmaidaaOGaemyBa02aaSbaaSqaaiabdQgaQbqabaaakeaacqWFdpWCdaqhaaWcbaGaem4AaSgabaGaeGOmaidaaOGaeiikaGIaemiEaGNaeiilaWIaemyEaKNaeiykaKIaey4kaSIae83Wdm3aa0baaSqaaiabd6gaUbqaaiabikdaYaaaaaGccqGGSaalaaa@5CF0@

where

σk2
 MathType@MTEF@5@5@+=feaafiart1ev1aaatCvAUfKttLearuWrP9MDH5MBPbIqV92AaeXatLxBI9gBaebbnrfifHhDYfgasaacH8akY=wiFfYdH8Gipec8Eeeu0xXdbba9frFj0=OqFfea0dXdd9vqai=hGuQ8kuc9pgc9s8qqaq=dirpe0xb9q8qiLsFr0=vr0=vr0dc8meaabaqaciaacaGaaeqabaqabeGadaaakeaaiiGacqWFdpWCdaqhaaWcbaGaem4AaSgabaGaeGOmaidaaaaa@30F4@(*x*,*y*) = max(0,σi2
 MathType@MTEF@5@5@+=feaafiart1ev1aaatCvAUfKttLearuWrP9MDH5MBPbIqV92AaeXatLxBI9gBaebbnrfifHhDYfgasaacH8akY=wiFfYdH8Gipec8Eeeu0xXdbba9frFj0=OqFfea0dXdd9vqai=hGuQ8kuc9pgc9s8qqaq=dirpe0xb9q8qiLsFr0=vr0=vr0dc8meaabaqaciaacaGaaeqabaqabeGadaaakeaaiiGacqWFdpWCdaqhaaWcbaGaemyAaKgabaGaeGOmaidaaaaa@30F0@(*x*,*y*) - σn2
 MathType@MTEF@5@5@+=feaafiart1ev1aaatCvAUfKttLearuWrP9MDH5MBPbIqV92AaeXatLxBI9gBaebbnrfifHhDYfgasaacH8akY=wiFfYdH8Gipec8Eeeu0xXdbba9frFj0=OqFfea0dXdd9vqai=hGuQ8kuc9pgc9s8qqaq=dirpe0xb9q8qiLsFr0=vr0=vr0dc8meaabaqaciaacaGaaeqabaqabeGadaaakeaaiiGacqWFdpWCdaqhaaWcbaGaemOBa4gabaGaeGOmaidaaaaa@30FA@)

and where *m*_*j *_is local mean, σi2
 MathType@MTEF@5@5@+=feaafiart1ev1aaatCvAUfKttLearuWrP9MDH5MBPbIqV92AaeXatLxBI9gBaebbnrfifHhDYfgasaacH8akY=wiFfYdH8Gipec8Eeeu0xXdbba9frFj0=OqFfea0dXdd9vqai=hGuQ8kuc9pgc9s8qqaq=dirpe0xb9q8qiLsFr0=vr0=vr0dc8meaabaqaciaacaGaaeqabaqabeGadaaakeaaiiGacqWFdpWCdaqhaaWcbaGaemyAaKgabaGaeGOmaidaaaaa@30F0@(*x*,*y*) is the image variance (computed on the template) and σn2
 MathType@MTEF@5@5@+=feaafiart1ev1aaatCvAUfKttLearuWrP9MDH5MBPbIqV92AaeXatLxBI9gBaebbnrfifHhDYfgasaacH8akY=wiFfYdH8Gipec8Eeeu0xXdbba9frFj0=OqFfea0dXdd9vqai=hGuQ8kuc9pgc9s8qqaq=dirpe0xb9q8qiLsFr0=vr0=vr0dc8meaabaqaciaacaGaaeqabaqabeGadaaakeaaiiGacqWFdpWCdaqhaaWcbaGaemOBa4gabaGaeGOmaidaaaaa@30FA@ is noise variance (defined *a priori*). The filter implies a tradeoff between smoothing efficiency and preservation of discontinuities.

In accordance with previous suggestion [15-17], only liver voxels were considered in the registration algorithm, since other abdominal organs may have motion relative to the liver [31] and thereby confound realignment. The liver is usually segmented to exclude uninteresting structures from the registration. We performed manual liver segmentation [15]: after selecting the slice with maximum area of liver, an irregular polygon (*mask*) was inserted manually to define the liver outline and exclude other tissues. The mask was then applied to all other slices in the volume. Upper and lower slices, where liver formed a small fraction of the total mask area, were excluded.

### Image registration

Liver motion was modeled by a rigid 3D translation to compensate for cranio-caudal movements and by a 2D local non-rigid registration to compensate for deformation within the slice plane.

Rigid registration results in rough realignment of the two volumes, producing slice-to-slice correspondence between the pre- and post-contrast datasets, while non-rigid registration locally refines the rigid realignment.

#### Rigid registration

Rigid 3D translation was achieved using normalized mutual information (NMI) as the intensity-based similarity measure [25]. NMI is defined as:

NMI=H (T(I1))+H(I2)H(T (I1),I2)     (1)
 MathType@MTEF@5@5@+=feaafiart1ev1aaatCvAUfKttLearuWrP9MDH5MBPbIqV92AaeXatLxBI9gBamXvP5wqSXMqHnxAJn0BKvguHDwzZbqegyvzYrwyUfgarqqtubsr4rNCHbGeaGqiA8vkIkVAFgIELiFeLkFeLk=iY=Hhbbf9v8qqaqFr0xc9pk0xbba9q8WqFfeaY=biLkVcLq=JHqVepeea0=as0db9vqpepesP0xe9Fve9Fve9GapdbaqaaeGacaGaaiaabeqaamqadiabaaGcbaGaemOta4Kaemyta0KaemysaKKaeyypa0ZaaSaaaeaacqWGibascaaMc8UaeiikaGIaemivaqLaeiikaGIaemysaK0aaSbaaSqaaiabigdaXaqabaGccqGGPaqkcqGGPaqkcqGHRaWkcqWGibascqGGOaakcqWGjbqsdaWgaaWcbaGaeGOmaidabeaakiabcMcaPaqaaiabdIeaijabcIcaOiabdsfaujaaykW7cqGGOaakcqWGjbqsdaWgaaWcbaGaeGymaedabeaakiabcMcaPiabcYcaSiabdMeajnaaBaaaleaacqaIYaGmaeqaaOGaeiykaKcaaiaaxMaacaWLjaWaaeWaaeaacqaIXaqmaiaawIcacaGLPaaaaaa@6128@

where *I*_1 _is the pre-contrast image, *I*_2 _the post-contrast image, *T *is the estimated translation and *T*(*I*_2_) is *I*_2 _corrected for the motion field; *H*(*I*_1_) and *H*(*T*(*I*_2_)) are the entropies of the images *I*_1 _and *T*(*I*_2_) respectively, and *H*(*I*_1_,*T*(*I*_2_)) is their joint entropy. In order to reduce computation time, only translations were considered. With the patient supine, rotations are negligible and anyway mainly occur around the z-axis [33]. Any rotations around this axis can be corrected in the subsequent 2D non-rigid registration step.

#### Non-rigid registration

**Figure 1:**
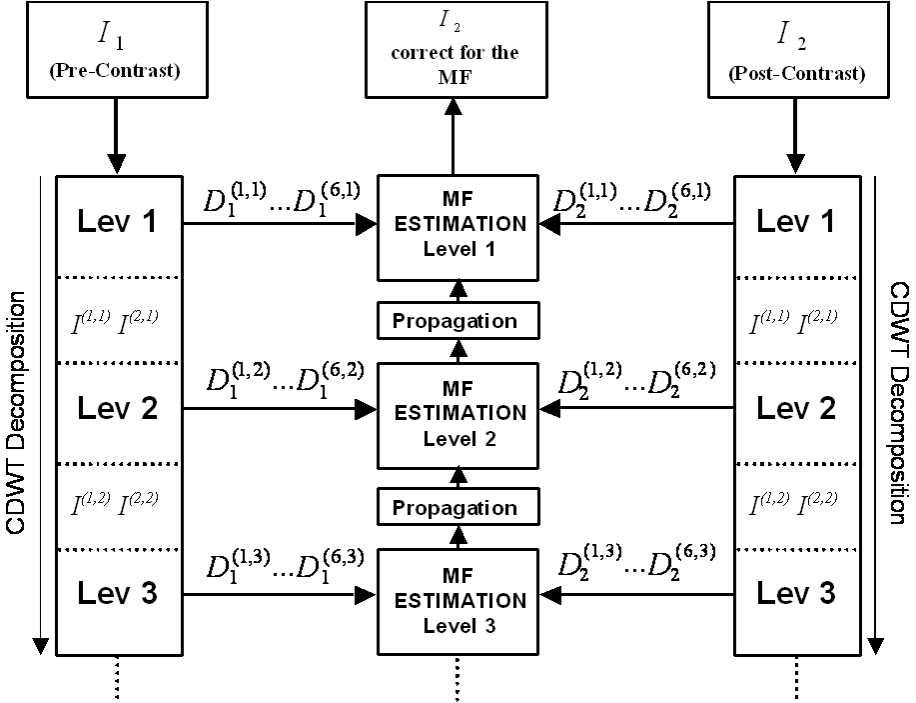
**Hierarchical algorithm structure**. CDWT decomposition is applied to the pre-contrast and the post-contrast images. At each level the CDWT produces 6 bandpass subimages per image that are then used to estimate the motion field (MF). The estimation starts at the coarsest level and progresses to the finest level.

The 2D non-rigid registration method relies on an adaptation of the algorithm of Magarey [26,27] originally applied for video coding. The algorithm is applied to each images pair of pre- and post-contrast volumes (Figure 1). There are four main steps in the algorithm.

#### (a) Image analysis

A CDWT pyramid is applied to the pre-contrast image (*I*_1_) and the post-contrast image (*I*_2_). The CDWT analyses each image *I*(**n**) using banks of *wavelet filters *Ψ(**n**) and *scaling filters *Φ(**n**) whose outputs are downsampled.

In particular, we have:

D(q,m)(n)=∑kI(k)ψ(q,m)(2mn−k)     (2)
 MathType@MTEF@5@5@+=feaafiart1ev1aaatCvAUfKttLearuWrP9MDH5MBPbIqV92AaeXatLxBI9gBaebbnrfifHhDYfgasaacH8akY=wiFfYdH8Gipec8Eeeu0xXdbba9frFj0=OqFfea0dXdd9vqai=hGuQ8kuc9pgc9s8qqaq=dirpe0xb9q8qiLsFr0=vr0=vr0dc8meaabaqaciaacaGaaeqabaqabeGadaaakeaacqWGebardaahaaWcbeqaaiabcIcaOiabdghaXjabcYcaSiabd2gaTjabcMcaPaaakiabcIcaOGqabiab=5gaUjabcMcaPiabg2da9maaqafabaGaemysaKKaeiikaGIae83AaSMaeiykaKccciGae4hYdK3aaWbaaSqabeaacqGGOaakcqWGXbqCcqGGSaalcqWGTbqBcqGGPaqkaaaabaGae83AaSgabeqdcqGHris5aOGaeiikaGIaeGOmaiZaaWbaaSqabeaacqWGTbqBaaGccqWFUbGBcqGHsislcqWFRbWAcqGGPaqkcaWLjaGaaCzcamaabmaabaGaeGOmaidacaGLOaGaayzkaaaaaa@5223@

I(p,m)(n)=∑kI(k)φ(p,m)(2mn−k)     (3)
 MathType@MTEF@5@5@+=feaafiart1ev1aaatCvAUfKttLearuWrP9MDH5MBPbIqV92AaeXatLxBI9gBaebbnrfifHhDYfgasaacH8akY=wiFfYdH8Gipec8Eeeu0xXdbba9frFj0=OqFfea0dXdd9vqai=hGuQ8kuc9pgc9s8qqaq=dirpe0xb9q8qiLsFr0=vr0=vr0dc8meaabaqaciaacaGaaeqabaqabeGadaaakeaacqWGjbqsdaahaaWcbeqaaiabcIcaOiabdchaWjabcYcaSiabd2gaTjabcMcaPaaakiabcIcaOGqabiab=5gaUjabcMcaPiabg2da9maaqafabaGaemysaKKaeiikaGIae83AaSMaeiykaKccciGae4NXdy2aaWbaaSqabeaacqGGOaakcqWGWbaCcqGGSaalcqWGTbqBcqGGPaqkaaaabaGae83AaSgabeqdcqGHris5aOGaeiikaGIaeGOmaiZaaWbaaSqabeaacqWGTbqBaaGccqWFUbGBcqGHsislcqWFRbWAcqGGPaqkcaWLjaGaaCzcamaabmaabaGaeG4mamdacaGLOaGaayzkaaaaaa@5216@

where Ψ(**n**) and Φ(**n**) are complex valued Gabor-like filters; *p *= 1,2 and *q *= 1, ..., 6, so that at each level there are eight complex outputs. *I*^(1,*m*) ^and *I*^(2,*m*) ^are images of lower resolution corresponding to inputs at the next level *m *+ 1. *D*^(*q*,*m*) ^are the downsampled bandpass subimages of *I*. Each wavelet filter, and its corresponding subimage, has a characteristic orientation specified by the spatial frequency **Ω**_*q*,*m*_. In the spatial frequency domain, the six filters cover the first and the second quadrants which contain non redundant information for image analysis.

#### (b) Multiresolution

At each level *m *of decomposition, the CDWT produces 6 oriented bandpass subimages, with resolution 1/2^*m *^times that of the original image. The algorithm starts estimation at the coarsest level *m*^max^, using the bandpass subimages to produce one motion estimate at each pixel. The field density is therefore 2mmax⁡
 MathType@MTEF@5@5@+=feaafiart1ev1aaatCvAUfKttLearuWrP9MDH5MBPbIqV92AaeXatLxBI9gBaebbnrfifHhDYfgasaacH8akY=wiFfYdH8Gipec8Eeeu0xXdbba9frFj0=OqFfea0dXdd9vqai=hGuQ8kuc9pgc9s8qqaq=dirpe0xb9q8qiLsFr0=vr0=vr0dc8meaabaqaciaacaGaaeqabaqabeGadaaakeaacqaIYaGmdaahaaWcbeqaaiabd2gaTnaaCaaameqabaGagiyBa0MaeiyyaeMaeiiEaGhaaaaaaaa@3382@, i.e. one vector per block of 2mmax⁡
 MathType@MTEF@5@5@+=feaafiart1ev1aaatCvAUfKttLearuWrP9MDH5MBPbIqV92AaeXatLxBI9gBaebbnrfifHhDYfgasaacH8akY=wiFfYdH8Gipec8Eeeu0xXdbba9frFj0=OqFfea0dXdd9vqai=hGuQ8kuc9pgc9s8qqaq=dirpe0xb9q8qiLsFr0=vr0=vr0dc8meaabaqaciaacaGaaeqabaqabeGadaaakeaacqaIYaGmdaahaaWcbeqaaiabd2gaTnaaCaaameqabaGagiyBa0MaeiyyaeMaeiiEaGhaaaaaaaa@3382@ × 2mmax⁡
 MathType@MTEF@5@5@+=feaafiart1ev1aaatCvAUfKttLearuWrP9MDH5MBPbIqV92AaeXatLxBI9gBaebbnrfifHhDYfgasaacH8akY=wiFfYdH8Gipec8Eeeu0xXdbba9frFj0=OqFfea0dXdd9vqai=hGuQ8kuc9pgc9s8qqaq=dirpe0xb9q8qiLsFr0=vr0=vr0dc8meaabaqaciaacaGaaeqabaqabeGadaaakeaacqaIYaGmdaahaaWcbeqaaiabd2gaTnaaCaaameqabaGagiyBa0MaeiyyaeMaeiiEaGhaaaaaaaa@3382@ input pixels. After interpolating by 2 in each direction, the estimation field is used as input to estimate at the next finer level, along with the 12 bandpass subimages at that level.

#### (c) Registration criterion

The principal part of the estimate of the motion field (MF) is the figure of merit based on the CDWT coefficients, known as the *subband squared difference *(*SSD*) and defined at each level as follows:

SSDm(n,f)=∑q=16|D1(q,m)(n+f)−D2(q,m)(n)|2P(q,m)     (4)
 MathType@MTEF@5@5@+=feaafiart1ev1aaatCvAUfKttLearuWrP9MDH5MBPbIqV92AaeXatLxBI9gBaebbnrfifHhDYfgasaacH8akY=wiFfYdH8Gipec8Eeeu0xXdbba9frFj0=OqFfea0dXdd9vqai=hGuQ8kuc9pgc9s8qqaq=dirpe0xb9q8qiLsFr0=vr0=vr0dc8meaabaqaciaacaGaaeqabaqabeGadaaakeaacqWGtbWucqWGtbWucqWGebardaahaaWcbeqaaiabd2gaTbaakiabcIcaOGqabiab=5gaUjabcYcaSiab=zgaMjabcMcaPiabg2da9maaqahabaWaaSaaaeaadaabdaqaaiabdseaenaaDaaaleaacqaIXaqmaeaacqGGOaakcqWGXbqCcqGGSaalcqWGTbqBcqGGPaqkaaGccqGGOaakcqWFUbGBcqGHRaWkcqWFMbGzcqGGPaqkcqGHsislcqWGebardaqhaaWcbaGaeGOmaidabaGaeiikaGIaemyCaeNaeiilaWIaemyBa0MaeiykaKcaaOGaeiikaGIae8NBa4MaeiykaKcacaGLhWUaayjcSdWaaWbaaSqabeaacqaIYaGmaaaakeaacqWGqbaudaahaaWcbeqaaiabcIcaOiabdghaXjabcYcaSiabd2gaTjabcMcaPaaaaaaabaGaemyCaeNaeyypa0JaeGymaedabaGaeGOnaydaniabggHiLdGccaWLjaGaaCzcamaabmaabaGaeGinaqdacaGLOaGaayzkaaaaaa@65F1@

where *D*_1_^(*q*,*m*)^(**n**+**f**) and *D*_2_^(*q*,*m*)^(**n**) are the CDWT coefficients at level *m *as obtained by decomposition of images *I*_1 _and *I*_2_, respectively, and where *P*^(*q*,*m*) ^is the energy of each wavelet filter. The *SSD *is a local measure, computed for each pixel **n**. It can be shown that for a small offset, a 2D quadratic model fits the *SSD *expression,

*SSD*^m^(**n**,**f**) ≈ 12
 MathType@MTEF@5@5@+=feaafiart1ev1aaatCvAUfKttLearuWrP9MDH5MBPbIqV92AaeXatLxBI9gBaebbnrfifHhDYfgasaacH8akY=wiFfYdH8Gipec8Eeeu0xXdbba9frFj0=OqFfea0dXdd9vqai=hGuQ8kuc9pgc9s8qqaq=dirpe0xb9q8qiLsFr0=vr0=vr0dc8meaabaqaciaacaGaaeqabaqabeGadaaakeaadaWcaaqaaiabigdaXaqaaiabikdaYaaaaaa@2E9E@(**f **- **f**_0_)^*T *^**κ**(**f **- **f**_0_) + *δ *    (5)

where the minimum value **f**_0 _is the motion estimate at the pixel **n**. The surface parameters {**f**_0_, **κ**, *δ*} (the minimum location, the *curvature matrix *and minimum height of the surface, respectively), are computed directly from the coefficients *D*_1_^(*q*,*m*)^(**n**+**f**), and *D*_2_^(*q*,*m*)^(**n**) and the spatial frequency **Ω**^*q*,*m*^. It turns out that **f**_0 _depends heavily on the phase of the two complex coefficient vectors, while the magnitudes have comparatively little influence. This property renders the algorithm insensitive to global perturbations such as level shifts and intensity scaling.

Note that it is possible to obtain **f**_0 _from equation (5) by analytical calculation, providing a very efficient way of computing **f **over a real interval of values, and avoiding the need for a time-consuming search over a discrete set of candidate solutions.

#### (d) Course to fine refinement

*SSD *is computed at each decomposition level. After estimation at the coarsest level (*m *= *m*^max^) motion estimates and other surface parameters used as starting values (using bilinear interpolation to double the field density) at the next finer level (*m *= *m*^max ^- 1). Hence the surfaces SSDmmax⁡−1
 MathType@MTEF@5@5@+=feaafiart1ev1aaatCvAUfKttLearuWrP9MDH5MBPbIqV92AaeXatLxBI9gBaebbnrfifHhDYfgasaacH8akY=wiFfYdH8Gipec8Eeeu0xXdbba9frFj0=OqFfea0dXdd9vqai=hGuQ8kuc9pgc9s8qqaq=dirpe0xb9q8qiLsFr0=vr0=vr0dc8meaabaqaciaacaGaaeqabaqabeGadaaakeaacqWGtbWucqWGtbWucqWGebardaahaaWcbeqaaiabd2gaTnaaCaaameqabaGagiyBa0MaeiyyaeMaeiiEaGhaaSGaeyOeI0IaeGymaedaaaaa@37E7@ are added to those from level *m*^max ^to give cumulative *SD *surfaces CSDmmax⁡−1
 MathType@MTEF@5@5@+=feaafiart1ev1aaatCvAUfKttLearuWrP9MDH5MBPbIqV92AaeXatLxBI9gBaebbnrfifHhDYfgasaacH8akY=wiFfYdH8Gipec8Eeeu0xXdbba9frFj0=OqFfea0dXdd9vqai=hGuQ8kuc9pgc9s8qqaq=dirpe0xb9q8qiLsFr0=vr0=vr0dc8meaabaqaciaacaGaaeqabaqabeGadaaakeaacqWGdbWqcqWGtbWucqWGebardaahaaWcbeqaaiabd2gaTnaaCaaameqabaGagiyBa0MaeiyyaeMaeiiEaGhaaSGaeyOeI0IaeGymaedaaaaa@37C7@. The *CSD *surfaces are then used as inputs to the next finer level *m*^max ^- 2, and the procedure is repeated until the required level of detail, given by *m*^min ^is reached. Finally the pre-contrast image is warped using the obtained motion field. A bilinear interpolation is used for non-integer locations.

## Results

### Quantitative performance

The new registration procedure was able to correct image movement to thereby produce improved subtraction images in all patients as determined by NMI assessment to compare pre- and post-contrast volumes.

**Figure 2:**
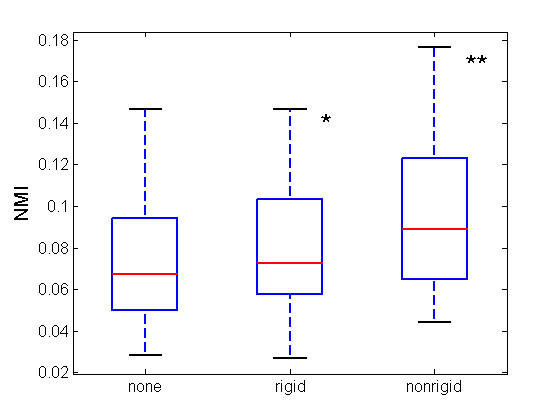
**NMI box plots**. Box-plots show NMI values for all 19 cases during the various phases of the registration algorithm. The red lines show increased median values after rigid (0.0673 vs 0.0726) and non-rigid registration (0.0726 vs 0.0891). (*n.s., **p < 0.001)

Figure 2 shows NMI values at prior to registration, after rigid registration and after non-rigid registration. NMI increased by about 8% after rigid registration (0.078 ± 0.031 vs. 0.073 ± 0.031, mean ± SD, n.s., paired *t*-test), and by a further 23% (0.096 ± 0.035 vs. 0.078 ± 0.031, p < 0.001, paired *t*-test) after non-rigid realignment. The overall increase was 31% compared to before registration. Rigid registration increased the NMI in 13/19 cases (thus, in 6 cases, the liver had not undergone cranio-caudal movement and the original volumes were already aligned), while non-rigid registration increased NMI in all cases.

### Clinical application

**Figure 3:**
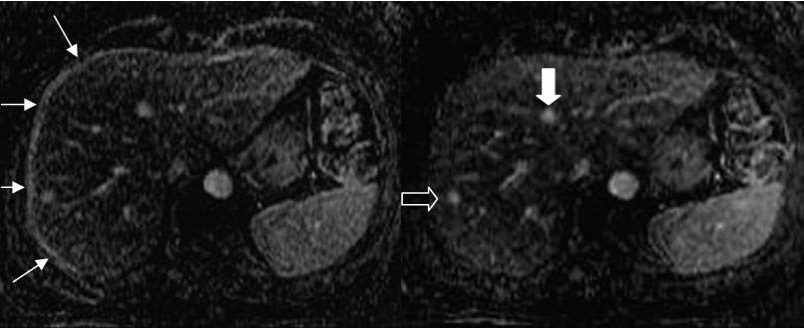
**Comparison between subtraction images**. Subtraction images before [left] and after [right] registration. In the right note the portal and hepatic veins, their course and the better identification of several anatomic details. Recognition of the enhancing lesion in 8^th ^segment is improved (open arrow in the right image). It is clear on the subtracted image after registration that the round zone of enhancement in 4^th ^segment is not a focal lesion but a branching portal ramus (white arrow in the right image). Finally, the high signal intensity artifact on the lateral and anterior margins of the liver disappears after registration (arrows in the left image).

Representative subtraction images before and after registration are shown in Figure 3. Note that after realignment, anatomic details are better visualized, the high signal intensity artifact at the edge of the liver has been eliminated, and enhanced structures (lesions and vessels) are better defined and not blurred by misalignment.

The diagnostic utility of subtraction images was scored by two expert radiologists in abdominal and liver MR imaging, by comparing registered subtraction images with the corresponding VIBE post-contrast images, and assigning a clinical score in the range 0–5, based the presence/absence of specific image characteristics such as third and fourth order portal rami, clarity of parenchyma, confidence of lesion identification, and minimun size of securely identified lesions. After registration, subtraction images had better clinical scores (3.20 ± 0.84 vs. 2.40 ± 0.55, p < 0.01, paired *t*-test) than VIBE post-contrast images. In all cases in subtraction images smaller lesions were more easily identified and small clusters of lesions were resolved into multiple adjoining small lesions.

**Figure 4:**
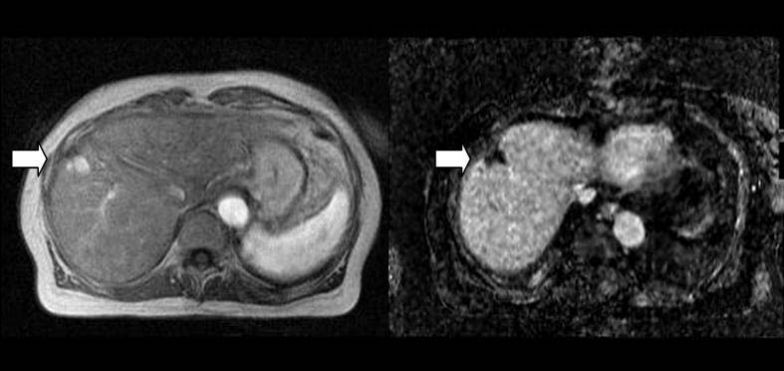
**HCC Relapse after treatment**. Tl-weighted 2D post-contrast image [left] and corresponding 3D subtraction image [right]. The arrow shows the lesion. The subtraction image shows complete wash-out indicating HCC (confirmed histologically). See text for further details.

Figure 4 shows a cirrhotic patient previously treated by percutaneous resection and percutaneous ablation of HCC and re-evaluated for suspected relapse. The Tl-weigthed 2D image in early arterial phase post-contrast shows early uptake by a plurinodular relapse (4a). The subtraction image of the same slice after registration shows early and complete lesion wash-out typical of nodular HCC relapse and shows no other areas of late uptake that would suggest A-V shunt or other post-treatment vascular anomalies. The site, size and number HCC nodules were confirmed at histology.

**Figure 5:**
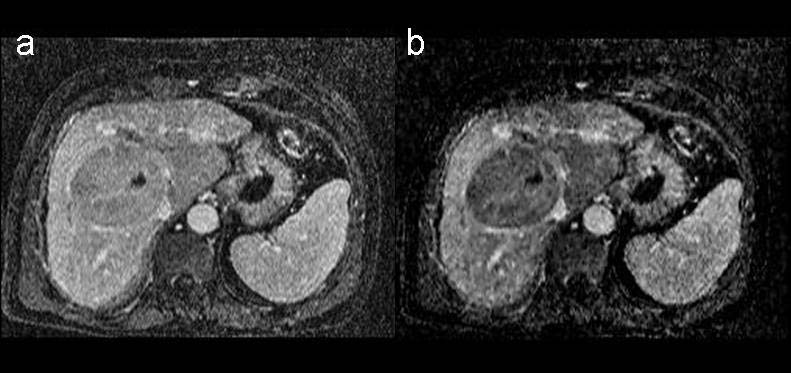
**Treated liver lesion**. Tl-weighted 3D post-contrast image [left] and corresponding 3D subtraction image [right]. The subtraction image shows complete absence of enhancement at the margins of the treated liver lesion that displaces the hepatic veins. Follow up confirmed absence of residual tumor. See text for further details.

In Figure 5 a treated liver lesion was evaluated by triphasic and late dynamic contrast-enhanced MRI. Post-treatment evaluation is vital for identifying minimal residual disease and thus deciding further management options. The portal phase 3D-VIBE post-contrast media image (5a) appears to show weak but diffuse lesion enhancement. The corresponding subtraction image after registration shows complete absence of enhancement at lesion center and margins. The displaced hepatic veins at the periphery of the lesion are well-demonstrated.

**Figure 6:**
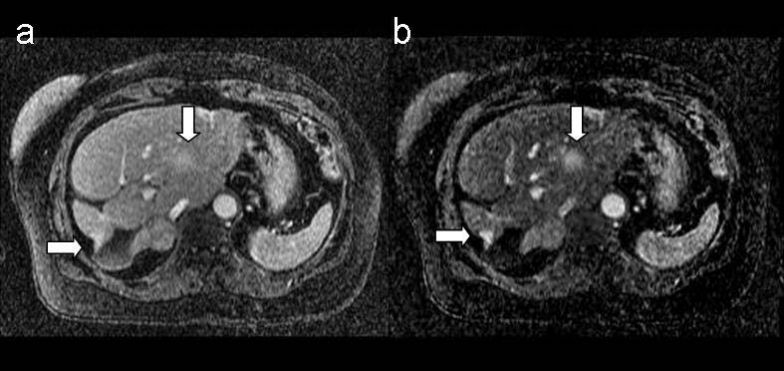
**Metastatic liver lesions from breast cancer**. Tl-weighted 3D post-contrast image [left] and corresponding 3D subtraction image, [right]. The arrows show metastatic lesions from breast cancer, better reveled in the subtraction image. See text for further details.

Figure 6 shows a dynamic contrast-enhanced image taken during follow-up of a patient with breast cancer. The 3D-VIBE image (6a) in late arterial-portal phase shows two inhomogeneous areas of weak contrast enhancement, with poorly defined borders in the parenchyma. These lesions are only suspicious for metastasis. In corresponding subtraction image after registration (6b) the lesions are more conspicuous, which uniform intense contrast uptake and sharper borders clearly indicating breast cancer metastasis. The lesions were biopsied and found to be metastatic breast cancer on histologic examination.

## Discussion and conclusion

We have presented and assessed a new approach to the registration of volumetric DCE-MR liver images. The method which realigns liver volumes using two procedures (rigid 3D translation followed by 2D non-rigid realignment using a CDWT algorithm) produced a significant increase of NMI (of about 31%). Subtraction images obtained after realignment showed, in comparison to VIBE post-contrast images, improved clinical utility as shown by clinical score.

Registration of hepatic volumes is not trivial because liver is a soft tissue undergoing considerable movement during respiration [18,20,33]. We approached the problem of hepatic volume realignment by a combination of rigid (only translations) and non-rigid registrations, as also suggested by other authors [17]-[20]. Rigid registration is usually employed to pre-align the two volumes (compensating for macroscopic liver movement in cranio-caudal direction) and aid the successive non-rigid registration to quickly converge to the optimal solution [21]. Non-rigid registration is usually based on maximization of NMI between two volumes and the transformation model is usually free-form 3D deformation defined on a control point grid (CPG) with cubic B-spline interpolation [18-20] or thin-plate spline interpolation [17]. Finally, a multiresolution strategy is usually employed to model both large displacement and small differences (following a coarse-to-fine strategy) and to improve the robustness and efficiency of registration. Although these approaches have been shown to be quite accurate [18,19], their main drawback is that they are time consuming. The performance of this registration method is also limited by the resolution of the CGP mesh, which is linearly related to computational complexity.

Improvements in computation time were obtained using a flexible framework [22] that permits non-uniform control point spacing to restrict deformation to localized regions of the image pair, excluding regions where the images are already in realignment or have been identified as rigid bodies.

In the approach we have developed, non-rigid 2D registration was performed using a CDWT algorithm with two main advantages: it is efficient for muliresolution analysis [26], and the CDWT coefficients contain, for each level, the information necessary for registration [27]. The motion field is obtained simply, pixel-by-pixel, as the minimum of a quadratic surface defined by the CDWT coefficients at each level. This results in the method being fast as there is no need for an exhaustive iterative search of the best solution. It has been estimated that registration normally requires 1.5 kflops/pixel [27]. In our Matlab implementation, registration of the entire liver (roughly 90 slices) took on average 3 minutes, running on a normal PC. It is likely that an implementation in C would be faster. Finally, all voxels contribute individually to defining the motion field, and hence registration does not require a priori definition of an elective CGP.

Our approach performs a multiresolution phase-based registration. It is therefore insensitive to the pixel intensity scale, shift, and additive Gaussian noise [26]. Although the CDWT algorithm does not maximize NMI directly, we found that NMI was significantly greater after realignment.

When rigid and non-rigid registration steps were compared we found, in agreement with a previous study [18], that substantial residual deformation often remained after rigid registration and an additional non-rigid approach was therefore required. Algorithms for the realignment of liver structures have been developed to merge MR and CT studies, emphasizing the enhancement of vascular structures for planning surgery [19] radiotherapy [16-18] or to quantitatively evaluate liver deformation [20,32,33]. Surprisingly, little attention has been paid to the application of registration techniques to produce improved subtraction images of the liver, although similar techniques have been developed for other organs [14,28]. Recently, dynamic subtraction MRI was used to characterize lesions in cirrhotic patients although small lesions were not shown optimally [13]. The authors concluded that misregistration limited the qualitative assessment of lesions <2 cm in size. Using our method subcentimetric lesions were frequently identified. We note finally that our algorithm was tested and validated on portal images only. Phase-based registration algorithms are insensitive to shifts in pixel intensity but they could fail on intensity-reversed images, such as those acquired during late portal phase or at equilibrium.

## Competing interests

The author(s) declare that they have no competing interests.

## Authors' contributions

LTM and PP conceived the study, developed the study protocol and the new method for dynamic subtraction of MR imaging of the liver. The search and study selection were conducted by PP and RM. Implementation and validation of the algorithm were conducted by KMP and AL. ES helped with the image analysis. LTM, PP and KMP contributed to the drafting of the manuscript. All authors have approved the content of the manuscript.

## Pre-publication history

The pre-publication history for this paper can be accessed here:
